# *Rhagoletis cerasi*: Oviposition Reduction Effects of Oil Products

**DOI:** 10.3390/insects5020319

**Published:** 2014-04-16

**Authors:** Claudia Daniel

**Affiliations:** Research Institute of Organic Agriculture (FiBL), Ackerstrasse 113, Postfach 219, CH-5070 Frick, Switzerland; E-Mail: claudia.daniel@fibl.org; Tel.: +41-0-62-865-72-72; Fax: +41-0-62-865-72-73

**Keywords:** Diptera, Tephritidae, organic agriculture, oviposition, behavior, horticultural oil, mineral oil

## Abstract

The European cherry fruit fly, *Rhagoletis cerasi* (L.) (Diptera: Tephritidae), is a highly destructive pest. Methods to control it are limited and alternatives are needed. Observations of cherry fruit flies suggest that females exert much effort to penetrate cherries at color change stage (from green to yellow) for oviposition. Therefore, the question arose as to whether a physical barrier on the fruit surface could reduce oviposition. The effects of different commercial horticultural oil products on *R. cerasi* oviposition were evaluated in a series of laboratory, semi-field and field experiments. In the laboratory experiments, the rate of successful oviposition on fruits treated with 0.25% v/v of the rapeseed oil product Telmion was significantly reduced by 90% compared to the untreated control. In semi-field experiments, deposits of 1% of rapeseed, mineral and paraffinic oil significantly reduced oviposition for up to 3 days. Semi-field experiments indicated that the oil products lose efficacy within 3 to 6 days after application due to degradation. Although treatments with the rapeseed oil product Telmion reduced infestation rates in an on-farm field experiment, the infested fruit clearly exceeded the level of market tolerance of 2%. Further research is needed to assess whether combinations of oil products, higher application rates and different formulations might improve field efficacy.

## 1. Introduction

The European cherry fruit fly, *Rhagoletis cerasi* (L.) (Diptera: Tephritidae), is a major pest of sweet cherries. *R. cerasi* is univoltine and overwinters as pupae in the soil [1]. The adult flies emerge in May and June [2,3] and begin to lay eggs about ten days after emergence [4,5]. The larvae develop inside the cherries [6]. Without insecticide treatment, up to 100% of fruit can be infested [7]. The low market tolerance for infested fruit is the principal reason for preventive insecticide treatments. The legal phase-out of insecticides threatens cherry production in Europe. For organic agriculture, a biocontrol strategy using the entomopathogenic fungus *Beauveria bassiana* in an oil-based, sprayable formulation (product Naturalis-L) was recently developed [8] and is currently registered for cherry fruit fly control in several European countries [9]. However, high production costs for the entomopathogenic fungus make this strategy more expensive than the use of conventional insecticides and alternative strategies are needed.

Oviposition behavior of cherry fruit flies is influenced by host fruit characteristics, such as size, shape [10], color [11], texture [12], surface structure [10], and chemosensory stimuli [12,13,14]. Cherries at the stage of color change from green to yellow, with a hardened cherry pit, and pulp at least 5 mm thick are preferred for oviposition [15]. Once a suitable fruit has been located, the female explores the surface structure for smoothness, softness and shape by walking in circles on the surface and decides whether to oviposit [10,16]. Fruit condition and chemistry might influence oviposition behavior during this exploration [17,18,19]. However, volatile odors of the oviposition site appear to be unimportant in eliciting oviposition [20,21], as females readily oviposit into inanimate objects [22], or fruits in which the larvae cannot complete their development [10,23]. Observing cherry fruit flies during oviposition gives the impression that much effort is required to penetrate the fruit [10]. Katsoyannos [12] noted that the duration of oviposition was six times longer in green and hard cherries than in fruit of optimal ripeness. Little is known about the physical force flies use to penetrate the skin.

Different authors have recently studied the use of physical barriers on the fruit surface to prevent oviposition of tephritid fruit flies [24,25,26,27,28,29,30,31,32,33]. Most research groups evaluated the effects of kaolin particle films. Kaolin impairs insect movement, feeding and other physical activities by the attachment of particles to the arthropods body [34,35]. Glenn *et al.* [34] observed that spirea aphids lost footing soon after being placed on particle film treated leaf surfaces due to particles clinging to their tarsae. However, treating cherries with kaolin would leave non-removable residues on the fruit. 

An alternative to kaolin might be the use of horticultural oil products. Oil products reduced oviposition in experiments with the Queensland fruit fly *Bactrocera tryoni* (Froggatt) (Diptera: Tephritidae) [28,29,30,33]. Nguyen *et al.* [30] observed that *B. tryoni* flies avoided treated fruits before landing and that they did not try to oviposit into oil treated fruit as soon as their tarsae came in contact with oil treated surfaces. They concluded that mineral oil volatiles repel females even before visual contact with fruit surfaces. This is contrary to the observations of Hidayat *et al.* [33], who showed that vegetable oils were not repellent for *B. tryoni* but acted as oviposition deterrent by creating a slippery layer on the fruit surface. 

In order to evaluate possible repellent or oviposition reduction effects of oil products on *R. cerasi* in sweet cherry, a series of experiments under laboratory, semi-field and field conditions was conducted. 

## 2. Materials and Methods

### 2.1. Laboratory Experiment

The laboratory experiment was conducted to evaluate the repellent or barrier effect of oil deposits on oviposition of *R. cerasi*. Flies were obtained from pupae collected from infested cherry trees in Basel-Land (north-western Switzerland). Flies were maintained under 16 h L:8 h D at a light intensity of 3000 lux and at 23 °C (day)/17 °C (night) and a relative humidity of 65%, as described by Boller [16] in transparent plastic cylinders with mesh covered top. Cages contained water and food prepared according to Boller [16] (4:1 mixture of sugar and yeast hydrolysate). Each cage contained two mature female flies and one male fly at the age of 25 days. To ensure a high oviposition pressure, the flies were not provided with any oviposition sites prior to the experiments.

Cherries of the variety Dollenseppler at the stage of color change from green to yellow were harvested from an untreated orchard (in Frick, North-Western Switzerland) from the shady northwest side of the tree canopy. For the experiment, 25 detached cherries were treated to run-off (4 mL per replicate) using hand-held, air-assisted spraying equipment (Devilbiss SRI 510, 1 bar) indoors. An untreated control was compared to the rapeseed oil product Telmion (Omya AG, Oftringen, Switzerland) and to the biocontrol product Naturalis-L (*Beauveria bassiana*, Intrachem Bio Italia S.p.A., Bergamo, Italy), which contains oil as a formulant. Both products were applied at a concentration of 0.25% v/v. Two hours after treatment, when the product coating had dried, cherries were exposed to *R. cerasi*. Four cherries were hung in the middle of each cage. Four cages (replicates) per treatment were installed. Cherries were changed daily and eggs were counted using a binocular microscope (6.3× magnification). The experiment was stopped after four days to avoid a direct effect of the entomopathogenic fungus *B. bassiana* on the flies. The behavior of flies towards treated and untreated cherries was observed in one cage per treatment, with special attention to landing and attempt at oviposition. Observations were conducted during one hour immediately before removing the cherries from the cages.

### 2.2. Semi-Field Experiments

The field persistence and degradation of oil formulations were investigated in two semi-field experiments. In the laboratory experiment described above, the flies were supplied with freshly treated fruit of the same stage daily. Therefore, the effects of fruit ripening, *i.e.*, increasing fruit surface and decreasing firmness of fruit, and degradation of the treatment film by sun or rain could not be evaluated. Semi-field experiments were conducted to clarify these points. Field collected *R. cerasi* were used for the semi-field experiments. Collection site, settings of the climate chamber, and experimental cages were the same as in the laboratory experiments described above. Each cage contained six female flies and two male flies. At the beginning of the first experiment, flies were 5–10 days old. For the second experiment, 14–17 day old flies were used. To ensure high oviposition pressure, flies were not provided with any oviposition sites prior to the experiments. Some flies (5%) died during the experiment and were replaced with flies of the same age.

In contrast to the laboratory experiment, treatments were not applied indoors, but on fruits on cherry trees. Each treatment was applied to run-off on two branches of one tree—one branch on the north side and one branch on the south side. Fruit samples were taken from these treated branches immediately (day 0) and three, six, and nine days after treatment. Two cherries—one from each side of the tree—were provided with water supply (small vial closed with cotton) and exposed to the flies in the laboratory for 48 h. Flies were kept for 24 h without oviposition sites before the next fruit were exposed. 

The semi-field experiment was replicated twice: in the first experiment cherries of the variety Star (application date: 18 May 2007, location Frick AG, altitude 410 m) at the stage of color change from green to yellow were used. In the second experiment cherries of the variety Schauenburger at the same stage (application date: 13 June 2007) from an orchard at a higher altitude (location Eptingen BL, altitude 680 m) were used. 

The experimental design of the first semi-field experiment comprised five cages (replicates) of the following seven treatments: untreated control, Telmion 0.3% (applied concentration 0.3%), Telmion 1% (applied concentration 1%), Genol Plant 1% (rapeseed oil, Syngenta Agro AG, Dielsdorf, Switzerland, applied concentration 1%), Mineral oil 1% (mineral oil, Omya AG, Oftringen, Switzerland, applied concentration 1%), Promanal 1% (paraffinic oil, W. Neudorff GmbH KG, Emmerthal, Germany, applied concentration 1%), and the formulants of Naturalis-L 1% (complete mixture formulants of Naturalis-L without conidia of *B. bassiana* obtained from Intrachem Bio Italia, applied concentration 1%). 

In the second semi-field experiment, Telmion was tested in combination with the pinolene products Heliosol and Nufilm to enhance UV-stability. The experimental design of the second semi-field experiment comprised five cages (replicates) of the following treatments: untreated control, Telmion 1% (rapeseed oil, applied concentration 1%), Telmion 1% mixed with Nufilm 0.1% (Pinolene, Intrachem Bio Italia S.p.A., Bergamo, Italy, applied concentration 0.1%), Telmion 1% mixed with Heliosol 0.1% (Pinolene, Omya AG, Oftringen, Switzerland, applied concentration 0.1%), Genol Plant 1% (rapeseed oil, applied concentration 1%), Mineral oil 1% (mineral oil, applied concentration 1%), and the formulants of Naturalis-L 1% (applied concentration 1%). The number of eggs was counted using a binocular microscope (6.3× magnification). 

### 2.3. Field Experiment

The efficacy of rapeseed oil treatments on fruit infestation under on-farm conditions was evaluated in the field experiment. The experiment was conducted in a commercial, organically managed orchard in north-western Switzerland (Eptingen BL) in 2006. This orchard consisted of 31 year old and 5 m high, semi-intensively managed standard cherry trees with an average yield of 15 to 25 kg per tree planted at intervals of 7 to 14 m in each direction. The experiment was arranged in a randomized design with four replicates (one tree per replicate and treatment; three replicates on the variety Schauenburger, one replicate on the variety Langstieler). Yellow sticky traps were the only control for *R. cerasi* used in this orchard in previous years.

The product Telmion was sprayed to run-off (15 L per tree) at a concentration of 1% using a commercial high-pressure hand-held gun. Untreated trees served as controls. Fly emergence was monitored daily by 24 photo-eclectors covering a surface area of 2 m^2^ each. Flight period and flight activity were monitored by yellow sticky traps (Rebell^®^amarillo, Andermatt Biocontrol AG, Grossdietwil, Switzerland). The first application was made on 21 June 2006, seven days after peak emergence (14 days after the beginning of emergence). Four days after the first application, 11.4 mm of rain occurred. Therefore, a second application was made on 26 June 2006. A third application was made on 3 July 2006 after 6.8 mm of rain. A random sample of 200 fruit per tree was taken immediately before the first application in order to estimate the initial infestation of cherries: The number of eggs and young larvae was counted using a binocular microscope (6.3× magnification). Fruit infestation was assessed at harvest on 11 July (variety Langstieler) and on 14 July (variety Schauenburger). A sample of 200 cherries was taken at random from each tree. Fruits were dissected under a binocular microscope to estimate the infestation level by *R. cerasi*. All larval instars and damaged fruits already abandoned by larvae for pupation were counted. 

### 2.4. Statistical Analysis

For the laboratory and semi-field experiment, the average number of eggs per female per day was calculated for each cage. Data were [log_10_(x + 1)] transformed. Normality of data and homogeneity of variance were tested before performing an ANOVA. The number of eggs per female and per day were analyzed by one-way ANOVA. Means were compared by Tukey HSD post hoc tests (α = 0.05). 

Data from field experiment were tested for normality of data and homogeneity of variance before performing an ANOVA. The infestation rate of cherries was analyzed by two-way ANOVA (treatment, variety).

## 3. Results

### 3.1. Laboratory Experiment

**Figure 1:**
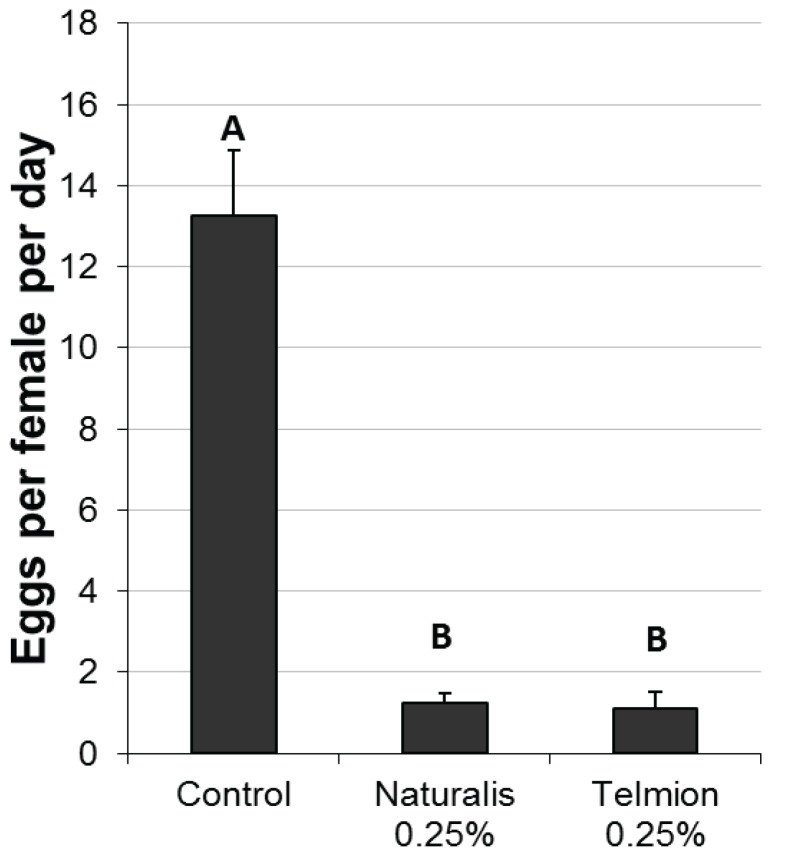
Oviposition reduction effect of Naturalis-L 0.25% and Telmion 0.25% in the laboratory experiment: Mean number of eggs per female per day (±se) in differently treated cherries. Different letters show significant differences (one-way ANOVA, Tukey HSD, α = 0.05).

Both treatments significantly reduced oviposition rates per female per day compared to the untreated control. No differences were found between the products Telmion and Naturalis-L (Figure 1, F_2,9_ = 53.18, *p* < 0.001). 

Observations of the behavior of the flies in the laboratory experiments showed that flies frequently landed on treated and untreated fruits and started their typical oviposition behavior. In untreated fruit, flies landed on the fruits, walked in circles to explore surface structure and chemistry, took position for penetration, penetrated, oviposited, started their typical post-oviposition host-marking behavior, and flew away from the fruit. If not disturbed by other flies, they needed 0.5 to 5 minutes to complete the whole sequence. However, behavior differed in treated fruit: flies landed, walked in circles, took position for penetration, tried to penetrate, their tarsae slipped on the oil treated surface, the flies adjusted their position again, tried to penetrate, stopped for cleaning of tarsae, took position for penetration again, tried to penetrate. Flies seemed not able to penetrate the skin with the ovipositor. Post-oviposition host-marking behavior was not observed on treated fruit. Flies usually did not leave the fruit until disturbed by other flies. Flies often spend 10 to 20 minutes on the same fruit.

### 3.2. Semi-Field Experiments

Climatic conditions differed between the two semi-field experiments: During the first semi-field experiment the weather was sunny (average temperature 19.2 °C, T_min_ = 6.5 °C, T_max_ = 30.0 °C; average global radiation per day: 6596 Wh/m^2^) and only slight precipitation (14 mm) occurred in the night from day three to day four. During the second semi-field experiment (average temperature 19.3 °C, T_min_ = 11.2 °C, T_max_ = 30.4 °C; average global radiation per day: 5376 Wh/m^2^) more rain fell: After the first fruit sampling and before sampling on the third day of the experiment, 52.9 mm of rain fell. Between the sampling on day three and on day six, another 38.1 mm of rain was recorded.

**Figure 2:**
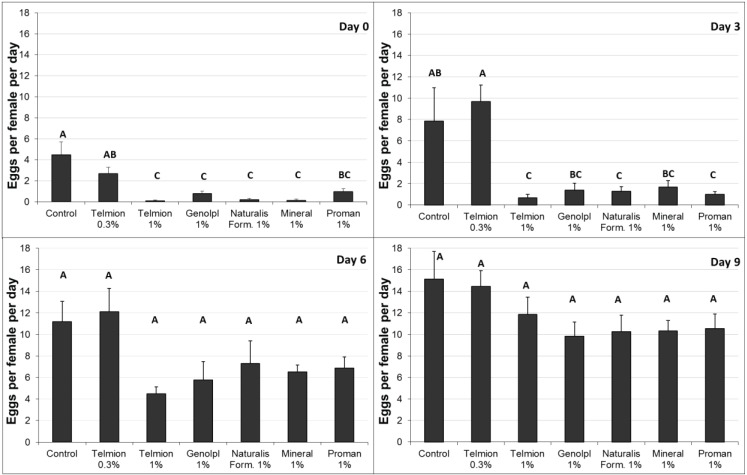
First semi-field experiment: Number of eggs per female per day (±se) in fruit samples exposed for oviposition immediately (0 day) and 3, 6 and 9 day after treatment. Different letters show significant differences (one-way ANOVA, Tukey HSD, α = 0.05).

In the first experiment (Figure 2), all treatments except Telmion 0.3% showed a significant reduction in the oviposition rate immediately after treatment (F_6,28_ = 16.54, *p* < 0.001). Three days after treatment, a significant effect was only observed for the treatments Telmion 1%, formulants of Naturalis-L 1%, and Promanal 1% (F_6,28_ = 8.43, *p* < 0.001). No significant differences were found between the different treatments six and nine days after treatments (F_6,28_ = 2.42, *p* = 0.052 and F_6,28_ = 1.40, *p* = 0.25, respectively). Promanal 1% caused phytotoxicity: oil droplets that remained on the surface after runoff caused small black spots on the lower end of the fruit. For this reason, Promanal was excluded in the second semi-field experiment.

**Figure 3:**
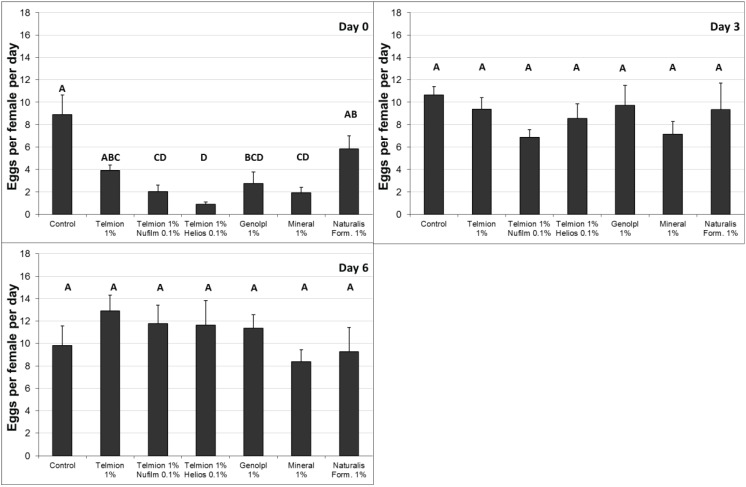
Second semi-field experiment: Number of eggs per female per day (±se) in fruit samples exposed for oviposition immediately (0 day), 3 and 6 day after treatment. Different letters show significant differences (one-way ANOVA, Tukey HSD, α = 0.05).

In the second semi-field experiment (Figure 3) the oviposition rate was significantly reduced immediately after treatment by all products except Telmion 1% and the formulates of Naturalis-L 1% (F_6,28_ = 10.54, *p* < 0.001). No differences were found between treatments three and six days after application (F_6,28_ = 0.78, *p* = 0.60 and F_6,28_ = 0.74, *p* = 0.62, respectively). Therefore the last examination at day 9 was omitted.

### 3.3. Field Experiment

**Figure 4:**
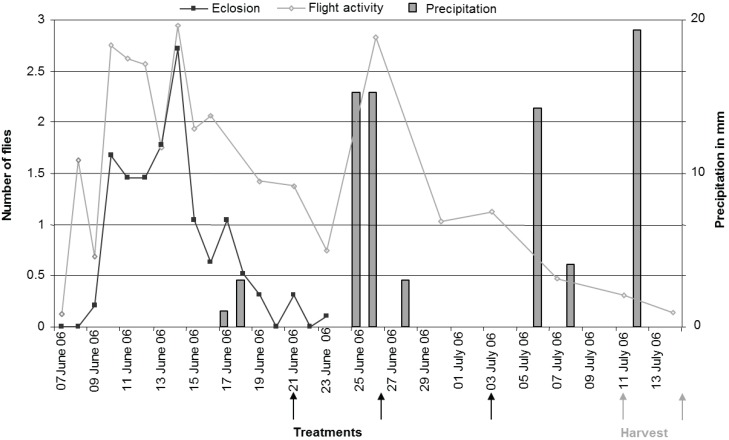
Field experiment: Eclosion of flies monitored by photo-eclectors (number of flies per 5 m^2^ per day); flight activity monitored by yellow sticky traps (number of flies per yellow sticky trap per day); dates of application and harvest as well as precipitation (mm) during the experimental period.

Figure 4 shows the eclosion of flies monitored by photo-eclectors, flight period monitored by yellow sticky traps, the dates of application of treatments and the dates of harvest as well as precipitation during the experimental period.

Fruit samples taken before the first application showed that 5.8% of the cherries already contained eggs. Infestation level at harvest was 41.5 ± 4.82% in the control trees and 27 ± 5.68% in the Telmion treated trees, but this difference was not significant (treatment: F_1,5_ = 4.56, *p* = 0.09; variety: F_1,5_ = 2.22, *p* = 0.20).

## 4. Discussion

Oil products are traditionally used against different stages of aphids or scale insects [36,37,38]. The mode of action was long supposed to be due to suffocation by blocking spiracles and tracheas of insects [39]. However, recent studies indicate more complicated effects on the integument, nervous and respiratory system of insects [40]. In addition, oil products can influence insect behavior acting as repellent, oviposition deterrent, or as physical barrier [28,29,30,33]. The experiments showed that oil products have an oviposition reduction effect on *R. cerasi*. The results might be related to several modes of action: (a) a physical barrier [33]; (b) suppression of attractant fruit volatiles [13]; (c) release of repellent volatiles from treated fruit [30]; (d) mimicry by oil molecules of naturally occurring repellent volatiles [41]; or (e) combination of these factors. 

In untreated fruit, fly behavior was consistent with literature [10,12,16]. In oil treated fruit, however, flies did not complete their typical oviposition behavior, but the repeated attempts to oviposit observed in treated fruits suggest that the oviposition reduction effect is mainly due to a physical barrier and not to the flies reaction to chemical cues of non-host volatiles, as observed for soy protein [41] and *Cornus*-extract treated fruits [13]. In their experiments with *B. tryoni*, Hidayat *et al.* [33] came to a similar conclusion: they also observed repeated landings and repeated attempts at oviposition in vegetable oil treated fruit, but flies were only able to penetrate fruits treated with lowest oil concentrations. They concluded that “the likely mechanism is that vegetable oils create a slippery surface, and females are unable to puncture the fruit skin for egg deposition” [33]. These observations are contrary to Nguyen *et al.* [30] who observed that *B. tryoni* flies avoided mineral oil treated fruits before landing and that they did not try to oviposit into oil treated fruit as soon as their tarsae came in contact with oil treated surfaces. They [30] concluded that mineral oil volatiles repel females even before visual contact with fruit surfaces. In their experiments, Hidayat *et al.* [33] observed a similar repellent effect of essential oils which they attribute to the volatile compounds of the essential oils. Further research is needed to investigate the mode of action of different oil products on oviposition behavior of tephritid flies.

For our laboratory experiment, mature females with high oviposition pressure were used. The experiment was stopped after four days to avoid a direct effect of the entomopathogenic fungus contained in the product Naturalis-L (*B. bassiana*) on the flies [42]. The rapeseed oil product Telmion and Naturalis-L showed similar efficacy. Thus, the observed effects of Naturalis-L are most likely due only to the repellent effect of the oil. The semi-field experiments, where only the formulants of the product Naturalis-L were applied, supported these findings: the formulants of Naturalis-L showed a similar efficacy like the other oil products. In view of these results it can be assumed that Naturalis-L has a dual mode of action: (1) some flies are killed due to fungus infection [8]; and (2) sub-lethally infected and weakened flies might be physically stressed by the oily film on the fruit surface to the point where they are unable to oviposit. 

In the first semi-field experiment in the Frick orchard, the reduction of efficacy seems mainly due to UV-mediated degradation of the products. During the second semi-field experiment, however, the loss of efficacy observed on the third day after treatment might be due to the wash-off of the treatment film by rain. The results of the semi-field experiments suggest that the effects of the different oil products are too short-lived to provide sufficient control of *R. cerasi* under sunny or rainy conditions. In addition, differences between the first and second semi-field experiment observed immediately after treatment (day 0) might also be due to the cherry variety or firmness of fruit: in the first semi-field experiment cherries of the variety Star were used, in the second semi-field experiment, cherries of the variety Schauenburger. In order to test the oil product Telmion under more realistic conditions, a field experiment was conducted in a commercial orchard on the varieties Schauenburger and Langstieler. 

Prior to the first application of 1% Telmion, fruit samples already showed an infestation rate of 5.8%. This was surprising, because the variety Schauenburger was only at the very beginning of color change from green to yellow. Moreover, the peak emergence was only seven days past and the pre-oviposition period of *R. cerasi* is considered to last about 10 days [43]. With an infestation rate of 42% in the untreated control at harvest, this initial infestation had probably only little influence on the results. Telmion treatments showed a tendency to lower the infestation level. However, the percent infestation (27%) in the Telmion treated trees exceeded the market tolerance of 2% by over a factor of ten.

## 5. Conclusions

In conclusion, oil products were shown to reduce oviposition by *R. cerasi*, by creating a physical barrier. However, oil deposits on the fruit surface degrade too rapidly to provide a good control of fruit infestation. Further research is needed to assess whether the combination of oil products with different formulants and adjuvants or higher application rates might improve field efficacy.
